# Limited value of platelet-related markers in diagnosing periprosthetic joint infection

**DOI:** 10.1186/s12891-023-07142-x

**Published:** 2024-01-02

**Authors:** Zhen-yu Song, Jin-cheng Huang, Dong-hui Wang, Qing-kai Wang, Jia-wei Feng, Qian-qian Cao, Xiao Chen, Zhi-peng Dai, Zong-yan Gao, Yi Jin

**Affiliations:** 1 Henan University People’s Hospital, Henan Provincial People’s Hospital, Zhengzhou, Henan China; 2 Department of Orthopaedics, Henan Provincial People’s Hospital, Henan University People’s Hospital, Zhengzhou University People’s Hospital, No. 7, Weiwu Road, Zhengzhou, 450003 Henan Province China

**Keywords:** Platelet, Diagnosis, Periprosthetic joint Infection, Platelet count to mean platelet volume ratio, Serum inflammatory markers

## Abstract

**Objective:**

To evaluate the diagnostic values of serum platelet count (PC), mean platelet volume ratio (MPV), platelet count to mean platelet volume ratio (PVR), platelet to lymphocyte ratio (PLR), platelet to neutrophil ratio (PNR), PC/Albumin-globulin ratio (PC/AGR), and PC/C-reactive protein (PC/ CRP) in the diagnosis of periprosthetic joint infection (PJI).

**Methods:**

The medical records were retrospectively analyzed of the 158 patients who had undergone hip or knee revisions from January 2018 to May 2022. Of them, 79 cases were diagnosed with PJI and 79 with aseptic loosening (AL). PJI was defined using the Musculoskeletal Infection Society criteria. The plasma levels of CRP, the erythrocyte sedimentation rate (ESR), PC, MPV, PVR, PLR, PNR, PC/AGR, and PC/CRP in the 2 groups were recorded and analyzed. In addition, tests were performed according to different joint types. The receiver operating characteristic curve was used to calculate the sensitivity and specificity of each indicator. The diagnostic value for each indicator was calculated according to the area under the curve (AUC).

**Results:**

The PC, PVR, PLR and PC/AGR levels in the PJI group were significantly higher than those in the AL group, while PC/CRP levels were significantly lower (*P* < 0.001). The AUC for PC/CRP, and PC/AGR was 0.804 and 0.802, respectively, which were slightly lower than that of CRP (0.826) and ESR (0.846). ROC analysis for PC/CRP, and PC/AGR revealed a cut-off value of 37.80 and 160.63, respectively, which provided a sensitivity of 73.42% and 84.81% and a specificity of 75.95% and 65.82% for PJI. The area under the curve of PLR and PC was 0.738 and 0.702. The area under the curve values for PVR, PNR, and MPV were 0.672, 0.553, and 0.544, respectively.

**Conclusions:**

The results of this study suggest that PC, PLR, PC/CRP, and PC/AGR values do not offer significant advantages over ESR or CRP values when employed for the diagnosis of PJI. PVR, PNR, and MPV were not reliable in the diagnosis of PJI.

## Introduction

Total joint arthroplasty (TJA) is a treatment option for end-stage hip and knee disease. Periprosthetic joint infection (PJI) is known as a catastrophic complication after prosthetic joint replacement, with an overall incidence of 1–2% [1, 2], which is a heavy burden for patients, physicians, and society. Combined with clinical practice, the diagnosis of PJI remains a considerable challenge due to the lack of a gold standard test for the diagnosis of PJI [3]. Two inflammatory markers, C-reactive protein (CRP) and erythrocyte sedimentation rate (ESR), have long been used to monitor disease activity in secondary infections and inflammatory diseases. The evaluation of CRP and ESR, two inflammatory markers, is included as one of the diagnostic criteria for PJI in the diagnostic guidelines developed by national organizations and is extensively used by physicians in the diagnosis of PJI, greatly improving the ability to diagnose PJI [4–7]. However, these two indicators are not specific to a particular disease. It has been found that overemphasis and reliance on CRP and ESR levels in PJI patients with hypovirulent bacterial infections can lead to misdiagnosis [8]. In addition, the levels of CRP and ESR were influenced by the different sexes, races, and ages of the subjects. For example, ESR level increased with age in women and African Americans, and this variation significantly impinged on the sensitivity, specificity, and accuracy of ESR and CRP in diagnosing PJI [9]. It has been reported that the detection of alpha-defensin levels by aspirating joint fluid has high accuracy in the diagnosis of PJI [10]. However, the most important thing is that carrying out this test requires medical institutions to have the corresponding detection equipment and technology, which greatly delays the diagnosis of PJI. Therefore, in the diagnosis of PJI, scholars continue to search for more convenient, reliable and stable blood-related markers.

In previous studies, researchers have discussed the diagnostic value of several coagulation-related parameters in the diagnosis of PJI, such as fibrinogen [11], fibrin degradation product (FDP) and fibrinolytic marker D-dimer [12], are strongly correlated with the infection status of PJI patients. The fibrinolytic marker D-dimer has been included in the definition of MSIS criteria diagnostic guidelines in 2018 providing a more comprehensive diagnostic strategy for the diagnosis of PJI [7], although the diagnostic value of D-dimer in the diagnosis of PJI is controversial [13].

Blood platelets are known to have a crucial role in the hemostasis and thrombosis, during immune responses [14]. Platelets are among the first cells to be recruited to sites of inflammation and infection, which play an instrumental role in initiating intravascular immune responses [15]. Blood platelets are directly participating in the antibacterial process as part of the body’s autoimmunity. When the body is in an infected state, Blood platelets can be activated by bacteria [16], which causes a reactively increased number of platelets [17]. Several studies have validated the idea that platelet count (PC) increases and mean platelet volume (MPV) decreases when the body is infected, thus providing a direction for the study of platelets and their related markers to predict the status of infection or inflammation in the body [18, 19]. In a recent study, Paziuk et al. [20] investigated the PC/MPV ratio as a diagnostic tool for PJI, which is a first in the literature. The diagnostic performance of platelets and platelet-related markers, such as PC, MPV, platelet count to mean platelet volume ratio (PVR), platelet to lymphocyte ratio (PLR), platelet to neutrophil ratio (PNR), PC/CRP and PC/Albumin-globulin ratio (PC/AGR), for the diagnosis of PJI remains controversial in the limited studies. Therefore, we aimed to investigate the diagnostic value of platelet-related markers PC, MPV, PVR, PLR, PNR, PC/CRP, and PC/AGR for PJI.

## Materials and methods

### Research design

This single-center retrospective cohort study encompassed hip and knee arthroplasty revision cases performed at our institution from January 2018 to May 2022. The study extracted patients who were diagnosed with either PJI or aseptic loosening (AL) to investigate the diagnostic efficacy of various biomarkers, including CRP, ESR, PC, MPV, PVR, PLR, PNR, PC/CRP, and PC/AGR, for PJI. Additionally, we conducted a subgroup analysis to evaluate the diagnostic value of these biomarkers specifically in the hip and knee subgroups. This study was approved by the Institutional Review Board (No: 2020.80).

### Inclusion and exclusion criteria

This study included patients who had undergone hip or knee revision surgery and were diagnosed with either PJI or aseptic loosening (AL) following total knee arthroplasty (TKA) or total hip arthroplasty (THA). Patients with periprosthetic fractures, joint dislocation and systemic inflammatory diseases (including inflammatory bowel disease, gout, sarcoidosis, multiple myeloma, rheumatoid arthritis, psoriasis, polymyositis or systemic lupus erythematosus) were excluded from the study. In addition, patients with incomplete clinical information will not be considered.

### Clinical definitions and data extraction

The definitive diagnosis of PJI and AL was determined based on the criteria defined in the 2014 MSIS for musculoskeletal infections [6] and the criteria for AL previously reported in the literature [13] (which are also the criteria we use in our clinical practice), respectively (Tables 1 and 2). Through our institution’s medical record system, we meticulously extracted the following patient data: demographics, symptoms and physical signs, diagnoses, and comorbidities, as well as laboratory test results including CRP, ESR, PC, MPV, lymphocyte, neutrophil and AGR.

**Table 1:** Definition of periprosthetic joint infection^†^

Diagnostic Criteria	Concrete Contents
Major Criteria	1) Two positive periprosthetic cultures with phenotypically identical organisms.
	2) A sinus tract communicating with the joint.
Minor Criteria	1) Elevated serum C-reactive protein (CRP) AND erythrocyte sedimentation rate (ESR);
	2) Elevated synovial fluid white blood cell (WBC) count OR + + change on leukocyte esterase test strip;
	3) Elevated synovial fluid polymorphonuclear neutrophil percentage (PMN%);
	4) Positive histological analyses of periprosthetic tissue;
	5) A single positive culture.

^†^According to the MSIS criteria, PJI is diagnosed when a patient has one of the two major criteria or three of the five minor criteria

**Table 2:** Definition of aseptic loosening of joint prosthesis

Evaluation criteria category	Concrete Contents
1. Clinical Symptoms	pain in the thigh or hip region, knee pain
2. Imaging Evaluation	radiological symptoms of loosening (disintegration of prosthesis components with the bone, displaced components of the prosthesis, circumferential radiolucent line)
3. cannot be defined as PJI	

### Measurement approaches

Fasting upper arm venous blood samples were collected from all patients on the morning of the second day after admission. Samples are sent to our clinical laboratory for sample testing within 1–2 h of collection. CRP was measured using special protein analyzer PA-990 (Sysmex, Japan). ESR was measured using Alifax TEST1 analyzer (Alifax, Italy). Routine blood examinations were performed on a Sysmex XN-9100 Automated Hematology System (Sysmex, Japan). The outcome data were collected from patients’ electronic medical records. The PLR, PNR, PVR, PC/CRP, and PC/AGR levels were calculated with the related parameters.

### Statistical analyses

The statistical analyses were performed with the IBM SPSS Statistics software (version 21). Normally distributed continuous data were shown as mean ± standard deviation (SD) and compared using student’s t-test. Non-normally distributed continuous data were shown as mean and compared using the Mann–Whitney U test. A *p* value < 0.05 was considered statistically significant. Receiver operating characteristic (ROC) curves were analyzed, assessing parameters such as sensitivity, specificity, the area under the ROC curve (AUC) and 95% confidence interval (CI). The Youden index (sensitivity + specificity − 1) was used to define optimal thresholds for PJI diagnosis. The discriminatory value of curves was interpreted as excellent (0.90-1.00), good (0.80–0.89), fair (0.70–0.79), poor (0.60–0.69), or failing (0.50–0.59).

## Results

A systematic search of our case system, based on clinical definitions and established inclusion and exclusion criteria, resulted in a total of 158 patients being included in this study, including 79 cases of PJI and 79 cases of AL, respectively. The baseline characteristics of the participants are shown in Table 3. There were no significant differences in demographic characteristics between these groups.

We assessed the levels of each biomarker in the PJI and AL groups. The results showed no significant difference in MPV and PNR levels between the two groups. For the rest of the indicators, the PC, PVR, PLR, and PC/AGR levels in the PJI group were significantly higher than those in the AL group, while PC/CRP levels were significantly lower (all *P* < 0.01). The CRP, ESR, PC/CRP, PC/AGR, PLR, PC, and PVR levels in the PJI group were 20.27 (interquartile range [IQR], 7.10, 42.20), 55.00 (29.00, 80.00), 13.88 (6.60, 54.42), 214.65 (172.50, 330.00), 188.06 (134.22, 248.23), 270.00 (217. 00, 322.00), and 27.86 (22.17, 37.23). CRP, ESR, PC/CRP, PC/AGR, PLR, PC, and PVR levels in the AL group were 1.70 (interquartile range [IQR], 0.50, 6.10) (*P* < 0.001), 15.00 (7.00,32.00) (*P* < 0.001), 115.29 (40.72, 350.00) (*P* < 0.001), 141.50 (112.11, 181.93) (*P* < 0.001), 128.99 (91.76, 166.47) (*P* < 0.001), 216.00 (179.00, 267.00) (*P* < 0.001), and 23.75 (17.67, 29.71) (*P* < 0.001) (Table 4). In addition, we assessed the levels of each biomarker separately, in the hip and knee subgroups (Tables 5 and 6).

**Table 3:** Patient demographics (N = 158)

Characteristic	PJI group (N = 79)	AL group (N = 79)	*P* value
Age, (years, mean ± SD)	66.00 ± 11.47	68.20 ± 10.52	0.210
Gender, n (%)			0.519
Male	35 (44.30%)	31 (39.24%)	
Female	44 (55.70%)	48 (60.76%)	
Affected joint			< 0.001
Hip, n (%)	31 (39.24%)	59 (74.68%)	
Knee, n (%)	48 (60.76%)	20 (25.32%)	

**Table 4:** Values of the tested biomarkers in the PJI and AL groups (N = 158), [M(P25, P75)]

Biomarker	PJI (N = 79)	AL (N = 79)	P*
CRP (mg/L)	20.27 (7.10, 42.20)	1.70 (0.50, 6.10)	< 0.001
ESR (mm/h)	55.00 (29.00, 80.00)	15.00 (7.00, 32.00)	< 0.001
PC/CRP	13.88 (6.60, 54.42)	115.29 (40.72, 350.00)	< 0.001
PC/AGR	214.65 (172.50, 330.00)	141.50 (112.11, 181.93)	< 0.001
PLR	188.06 (134.22, 248.23)	128.99 (91.76, 166.47)	< 0.001
PC (× 10^9^/L)	270.00 (217. 00, 322.00)	216.00 (179.00, 267.00)	< 0.001
PVR	27.86(22.17, 37.23)	23.75 (17.67, 29.71)	< 0.001
PNR	74.24 (51.32, 90.67)	62.83 (48.65, 84.23)	0.246
MPV	9.60 (8.50, 10.30)	9.70 (8.60, 10.70)	0.358

**Table 5:** Biomarker Values in PJI and AL Groups (Hip Joint Subgroup, N = 90), [M(P25, P75)]

Biomarker	PJI (N = 79)	AL (N = 79)	P*
CRP (mg/L)	22.50 (9.37, 42.20)	1.70 (0.50, 7.40)	< 0.001
ESR (mm/h)	68.00 (34.00, 91.00)	16.00 (6.00, 36.00)	< 0.001
PC/CRP	14.07 (5.92, 27.53)	120.66 (27.84, 367.74)	< 0.001
PC/AGR	209.00 (169.38, 351.79)	139.29 (107.13, 178.26)	< 0.001
PLR	196.45 (135.12, 261.18)	131.21 (92.54, 173.62)	0.004
PC (× 10^9^/L)	270.00 (217.00, 332.00)	213.00 (186.00, 271.00)	< 0.001
PVR	27.16(23.98, 35.25)	23.21 (16.90, 29.71)	< 0.001
PNR	62.39 (46.89, 83.24)	62.83 (49.00, 83.13)	0.956
MPV	9.90 (8.70, 10.50)	9.90 (8.60, 10.90)	0.677

**Table 6:** Biomarker Values in PJI and AL Groups (Knee Joint Subgroup, N = 68), [M(P25, P75)]

Biomarker	PJI (N = 79)	AL (N = 79)	P*
CRP (mg/L)	19.35 (4.82, 47.65)	2.05 (0.53, 3.52)	< 0.001
ESR (mm/h)	54.50 (29.00, 77.75)	13.50 (8.25, 20.25)	< 0.001
PC/CRP	13.86 (6.66, 63.40)	106.52 (56.21, 334.50)	< 0.001
PC/AGR	217.94 (172.51, 329.61)	145.74 (118.02, 183.95)	< 0.001
PLR	181.39 (133.07, 239.56)	122.41 (77.68, 159.30)	0.001
PC (× 10^9^/L)	265.50 (211.00, 321.75)	227.00 (170.50, 265.00)	0.028
PVR	28.00 (21.56, 39.44)	24.77 (19.28, 30.75)	0.073
PNR	77.22 (51.36, 99.45)	63.05 (45.10, 93.01)	0.282
MPV	9.55 (8.25, 10.00)	9.10 (8.55, 10.18)	0.952

**Fig. 1:**
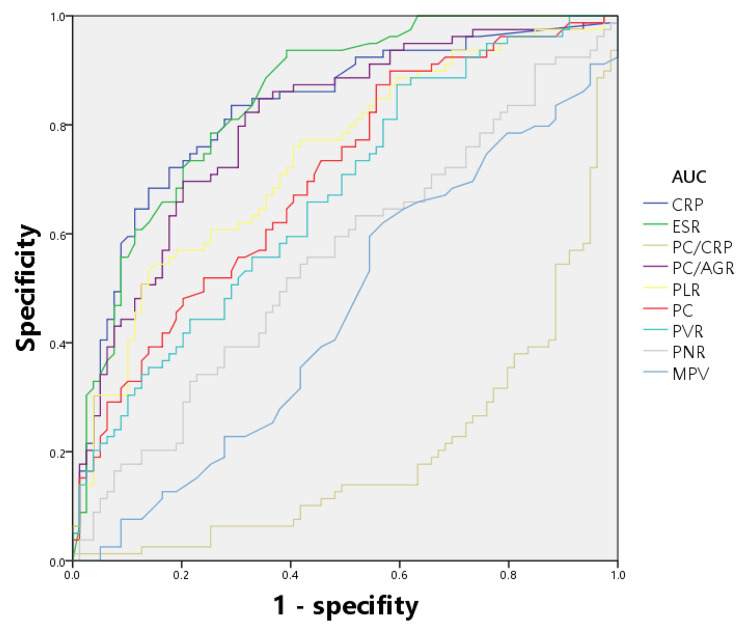
ROC analysis of all parameters

**Fig. 2:**
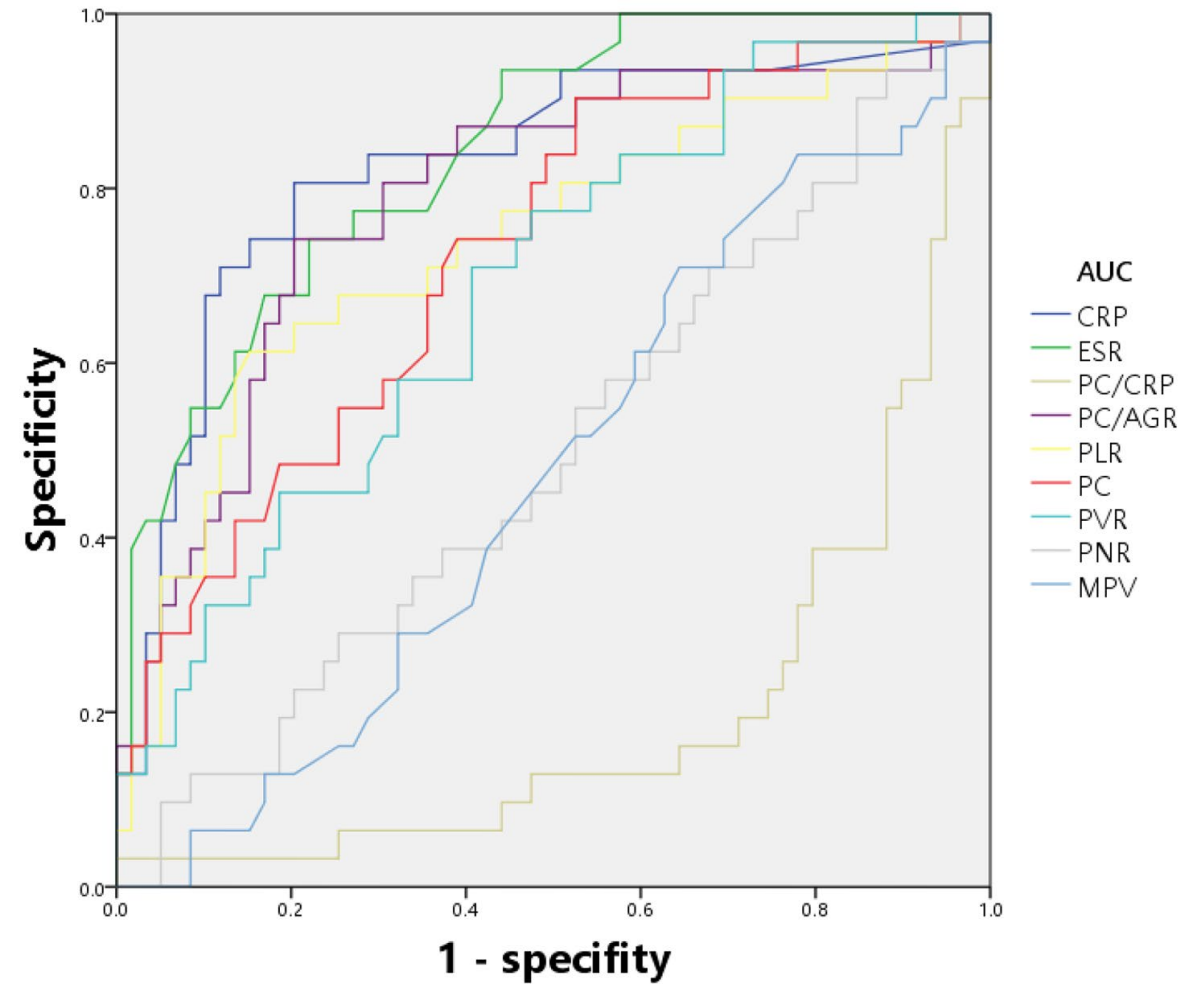
ROC analysis of all parameters (Hip Joint Subgroup)

**Fig. 3:**
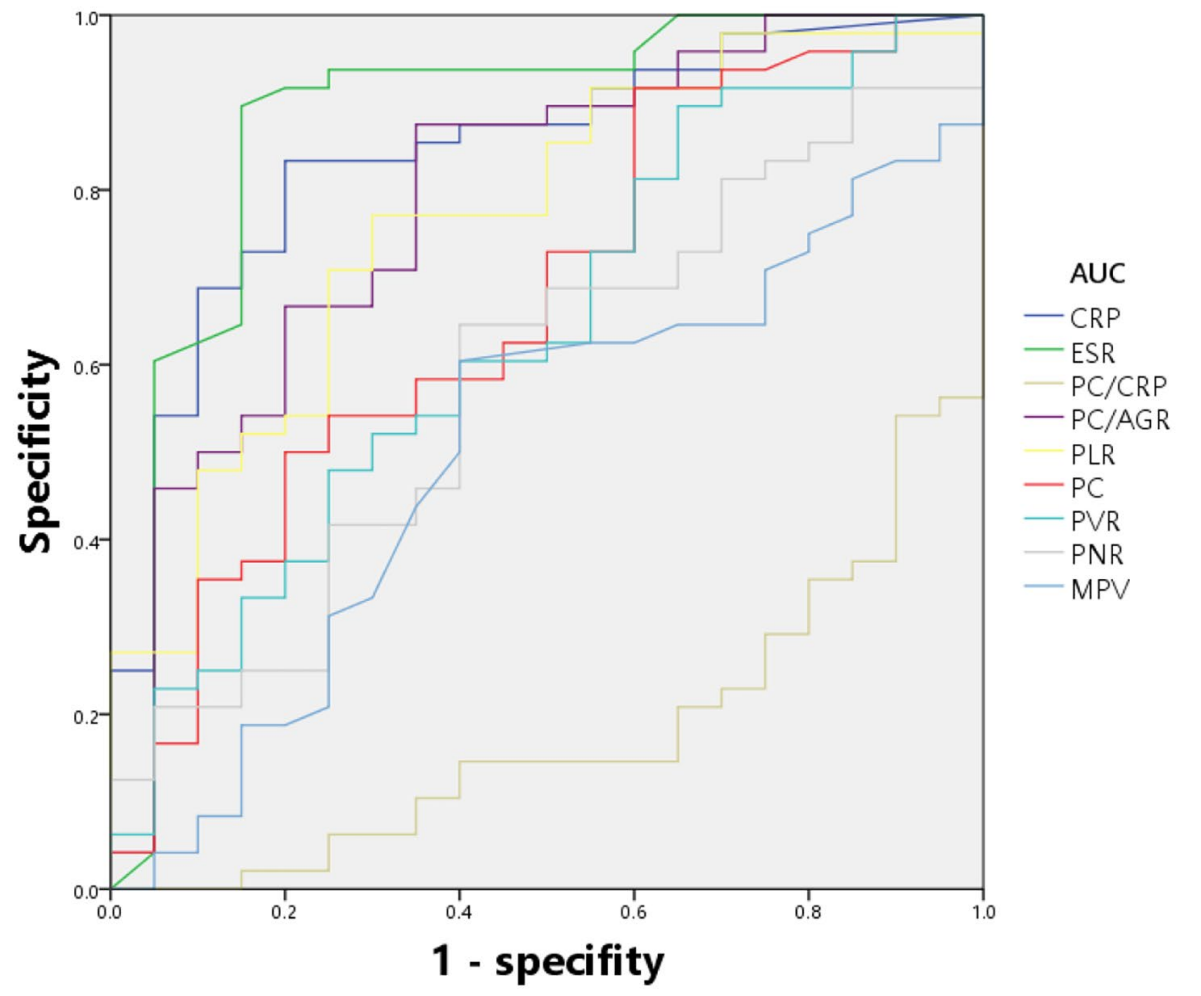
ROC analysis of all parameters (Knee Joint Subgroup)

All tested markers were evaluated and depicted in a receiver operating characteristic curve (Fig. 1). The AUCs for CRP, ESR, PC/CRP, and PC/AGR were 0.826 (95% confidence interval (CI), 0.758–0.882), 0.846 (0.780–0.898), 0.804(0.733–0.863), and 0.802 (0.731–0.861), respectively. The AUC of these four indicators ranged from 0.800 to 0.899, indicating that they are of good value for the diagnosis of PJI. The AUCs for PLR and PC were 0.738 (0.662–0.804), 0.702 (0.624–0.772), and the value of the curves was classified as fair. The indicator with the lowest value for diagnosing PJI was MPV, which had an AUC of 0.544 (0.463–0.623), indicating that the diagnostic effect of PJI was failing. The best prediction threshold for PC/CRP diagnosis of PJI was 37.80 (sensitivity: 73.42%, specificity: 75.95%). The best prediction cut-off value for PC/AGR was 160.63 (sensitivity: 84.81%, specificity: 65.82%). ROC analysis for PLR revealed a cut-off value of 181.51, which provided 54.43% sensitivity and 84.81% specificity for PJI (Table 7; Fig. 1). Additionally, we conducted separate evaluations of the diagnostic value of each biomarker for PJI in the hip and knee subgroups (Tables 8 and 9), and we depicted the ROC curves for each subgroup (Figs. 2 and 3).

**Table 7:** The diagnostic performance of different serum parameters for PJI

Biomarker	AUC (95%CI)	Youden index	Optimal cutoff value	Sensitivity (%)	Specificity (%)
CRP (mg/L)	0.826(0.758–0.882)	0.5443	3.60	83.54	70.89
ESR (mm/h)	0.846(0.780–0.898)	0.5443	19.00	93.67	60.76
PC/CRP	0.804(0.733–0.863)	0.4937	37.80	73.42	75.95
PC/AGR	0.802(0.731–0.861)	0.5063	160.63	84.81	65.82
PLR	0.738(0.662–0.804)	0.3924	181.51	54.43	84.81
PC (× 10^9^/L)	0.702(0.624–0.772)	0.3165	203.00	89.87	41.77
PVR	0.672(0.593–0.744)	0.2785	20.47	87.34	40.51
PNR	0.553(0.472–0.632)	0.1266	69.28	55.70	56.96
MPV	0.544(0.463–0.623)	0.1139	10.20	74.68	36.71

**Table 8:** The diagnostic performance of different serum parameters for PJI (Hip Joint Subgroup)

Biomarker	AUC (95%CI)	Youden index	Optimal cutoff value	Sensitivity (%)	Specificity (%)
CRP (mg/L)	0.826(0.732–0.898)	0.6031	9.11	80.65	79.66
ESR (mm/h)	0.842(0.750–0.911)	0.5216	37.00	74.19	77.97
PC/CRP	0.804(0.707–0.880)	0.5522	37.8	80.65	74.58
PC/AGR	0.791(0.693–0.870)	0.5385	182.19	74.19	79.66
PLR	0.741(0.637–0.827)	0.4604	181.51	61.29	84.75
PC (× 10^9^/L)	0.726(0.621–0.814)	0.3778	208.00	90.32	47.46
PVR	0.684(0.578–0.778)	0.3029	24.52	70.97	59.32
PNR	0.504(0.396–0.611)	0.0574	88.78	87.10	18.64
MPV	0.527(0.419–0.633)	0.1099	10.70	83.87	27.12

**Table 9:** The diagnostic performance of different serum parameters for PJI (Knee Joint Subgroup)

Biomarker	AUC (95%CI)	Youden index	Optimal cutoff value	Sensitivity (%)	Specificity (%)
CRP (mg/L)	0.845(0.745–0.946)	0.6333	3.60	83.33	80.00
ESR (mm/h)	0.882(0.773–0.990)	0.7458	22.00	89.58	85.00
PC/CRP	0.822(0.721–0.923)	0.5250	20.60	62.50	90.00
PC/AGR	0.792(0.667–0.916)	0.5250	160.63	87.50	65.00
PLR	0.768(0.645–0.891)	0.4708	132.57	77.08	70.00
PC (× 10^9^/L)	0.670(0.525–0.816)	0.3167	184	91.67	40.00
PVR	0.639(0.491–0.786)	0.2458	19.89	89.58	35.00
PNR	0.583(0.436–0.730)	0.2458	67.13	64.58	60.00
MPV	0.505(0.353–0.656)	0.2042	9.10	60.42	60.00

## Discussion

As a catastrophic complication after artificial joint replacement, PJI poses many difficulties for patients and physicians in terms of diagnosis and treatment. According to statistical results, the number of arthroplasty procedures is increasing yearly. In this context, this serious complication is expected to increase rapidly [17]. Therefore, timely diagnosis of PJI by clinicians and the development of the correct treatment plan as soon as possible will not only substantially reduce patient suffering, but also significantly lower the cost of the entire treatment phase [21]. Thus, an accurate diagnosis of infection is critical. Although new tools for the diagnosis of PJI such as Calprotectin, Presepsin, and Neopterin are being discovered with the development and application of new technologies [22]. However, the diagnostic efficacy of these new diagnostic markers and their feasibility for wider application is still a matter of debate. In addition, polymerase chain reaction (PCR) based pathogen detection techniques [23, 24], such as metagenomic next-generation sequencing (mNGS) and pathogen-targeted next-generation sequencing (ptNGS), are being used in the diagnosis and treatment of PJI. These new technologies offer benefits to patients with negative culture results. Consequently, Many researchers strive for new markers to predict PJI.

Platelets are involved in coagulation and inflammatory responses and have a vital role in the microenvironment of infections and tumours [17, 25]. Tumour cells or inflammatory cells stimulate megakaryocytes by releasing inflammatory mediators, which causes an increasing number of platelets. When the body is in an infected state, platelets are affected by bacterial activation in increased numbers and produce antimicrobial peptides [26], while MPV is reduced due to high concentrations of thrombopoietin in megakaryocytes [27]. Therefore, scholars have discussed the association of PC, MPV, and PVR with infectious and inflammatory conditions such as sepsis [28], febrile seizure [29], peritonitis, and pancreatitis [30]. Xu et al. [31] showed that the AUC value of PC for diagnosing PJI was 0.746, and the sensitivity and specificity were respectively 57.5% and 83.1%. Compared to the results of this study, the value of PC in the diagnosis of PJI is fair in the diagnostic value according to the criteria, when 203 × 10^9^/L is used as the sensitivity and specificity of the optimal cut-off value were 89.87% and 41.77%, respectively, and the AUC value was 0.702. In this study, PC was found to have high sensitivity and low specificity in the diagnosis of PJI, contrary to the sensitivity, specificity results concluded by Xu et al. Mean platelet volume (MPV) is an indicator of platelet size and a marker of platelet activity. New research has found that the platelet-related biomarker mean platelet volume (MPV) is associated with PJI [32], but the results of this study demonstrated that MPV had the lowest ability to independently diagnose PJI in this study (AUV = 0.554) and did not have diagnostic value for PJI. Based on the opposite trends of PC and MPV in the infected state, scholars have explored the diagnostic value of the PC/MPV ratio (PVR) for PJI. Klemt et al. [18] explored the diagnostic value of PVR in PJI and showed that at a threshold of 27.8, the area under the PVR display curve was 0.86, with a sensitivity and specificity of 86.4% and 75.5%, respectively, and a diagnostic efficacy rating of good. Compared to the results of this study, at the threshold of 20.47, the PVR showed an area under the curve of 0.672, with a sensitivity and specificity of 87.34% and 40.51%, respectively, and a diagnostic performance rating of poor. The different evaluation results of the two studies indicated the controversial nature of PVR in diagnosing PJI and the need for more studies to validate PVR’s ability to diagnose PJI. In this study, PC (89.87%) and PVR (87.34%) were slightly more sensitive than CRP (83.54%), but significantly less than CRP (70.89%) and ESR (60.76%), resulting in higher AUCs for CRP and ESR than for PC and PVR.

Platelets activated by bacterial stimulation sense the pathogens invading the body through specific receptors and cause leukocytes, neutrophils and other immune cells to regulate at the site of infection and inflammation [33]. Newly published research has focused on platelet binding to inflammatory cells as a new biomarker for exploring the value of these parameters in inflammatory, infectious, and neoplastic diseases. In a recent study, Pociute et al. [34] found PNR, and PLR to be promising in the diagnosis of serious bacterial infection (SBI) and sepsis in early pediatric. Ozer et al. [35] reported that the PLR levels could be used to assess subclinical inflammation (SI) in familial mediterranean fever (FMF). Marta et al.‘s study explored the prognostic value of the Immune Inflammatory Biomarker PLR in breast cancer patients treated with neoadjuvant chemotherapy [36]. In terms of the diagnostic value of PJI, Klemt et al. [18] showed that the PLR level, at a threshold of 237.9, had an area under the PVR display curve of 0.86, with a sensitivity and specificity of 75.92% and 82.78%, respectively, and a diagnostic evaluation of good. Compared with the results of this study, at a cutoff point of 181.51, PVR demonstrates an area under the curve of 0.738, with a sensitivity and specificity of 54.43% and 84.81%, respectively, and a diagnostic evaluation of fair. In this study, PLR demonstrated high specificity in its ability to diagnose PJI, while lower sensitivity resulted in lower AUC values than Christian Klemt’s results. This also implies that PLR is controversial in diagnosing PJI and more studies are needed to validate the diagnostic ability of PLR. When the organism is in an infected state, immunoglobulins are elevated and albumin levels are decreased. Therefore, based on the opposite trend of albumin and globulin, researchers found that low AGR levels were closely related to the prognosis and diagnosis of PJI [37]. Shang et al. [38] proposed to combine PC with AGR and use PC/AGR as a new diagnostic parameter for the diagnosis of PJI with good results. The diagnostic value of PC/AGR for PJI was likewise discussed in this study, and it was found that at a threshold of 160.63, PVR showed an area under the curve of 0.802, with a sensitivity and specificity of 84.81% and 65.82%, respectively, and a good diagnostic efficacy rating, which was the highest among all indicators. In addition, we explored the diagnostic value of PC/CRP for PJI for the first time and found that it had an AUC of 0.804, a sensitivity and specificity of 73.42% and 75.95%, respectively.

In conclusion, the diagnostic power of platelet-related markers in the diagnosis of PJI needs to be further explored. Despite our further analyses, platelet-related markers did not demonstrate excellent diagnostic performance in the hip and knee subgroups. We also examined different studies exhibiting different evaluation results for the same marker. The discrepancy in results may be related to the race of the included studies and the differences in the inclusion and exclusion criteria developed. In addition, the different diagnoses derived for a very small number of patients using different versions of the PJI diagnostic guidelines in the various studies could also have had an impact on the results of this study. The diagnostic value of platelet-related markers for PJI caused by low-virulence pathogens needs to be further explored, which can help to develop timely treatment plans for patients with acute and chronic PJI. Although the diagnostic value of platelet-related indicators for PJI is not superior to the traditional inflammatory markers CRP and ESR, it does not cause additional financial burden and physical pain to patients in the admission screening of hip and knee revision patients. As such, there is great benefit in exploring the diagnostic efficacy of platelet-related markers for PJI to provide an adjunctive diagnosis for patients.

### Limitations

The study has several limitations. Firstly, this was a retrospective study, and although most cases are well documented, there can be inaccurate information in medical records. Secondly, we did not perform a clear categorical study of patients with a history of aspirin administration 5–7 days before surgery, which may have influenced the results. Additionally, due to PJI and AL of the low incidence, the number of cases in this study is limited. Moreover, the optimal threshold for platelet-related markers to diagnose PJI still needs to be further investigated because of the different detection instruments and different detection results. Lastly, the present cohort were all from patients of the same ethnicity, so our results should be interpreted cautiously when branched out to other ethnic groups. Therefore, a well-designed, multicenter, large-sample study is crucial to further confirm our findings.

## Conclusions

The results of this study suggest that PC, PLR, PC/CRP, and PC/AGR values do not offer significant advantages over ESR or CRP values when employed for the diagnosis of PJI. PVR, PNR, and MPV were not reliable in the diagnosis of PJI. However, multicenter and larger sample size studies are essential to confirm and extend these results.

## Data Availability

The data that support the findings of this study are available on request from the corresponding author.

## Abbreviations

PJI: periprosthetic joint infection
AL: aseptic loosening
CRP: C-reactive protein
ESR: erythrocyte sedimentation rate
PC: platelet count
MPV: mean platelet volume ratio
PVR: platelet count to mean platelet volume ratio
PLR: platelet to lymphocyte ratio
PNR: platelet to neutrophil ratio
